# Imaging of Acute Complications of Community-Acquired Pneumonia in the Paediatric Population—From Chest Radiography to MRI

**DOI:** 10.3390/children11010122

**Published:** 2024-01-18

**Authors:** Efthymia Alexopoulou, Spyridon Prountzos, Maria Raissaki, Argyro Mazioti, Pablo Caro-Dominguez, Franz Wolfgang Hirsch, Jovan Lovrenski, Pierluigi Ciet

**Affiliations:** 1 2nd Department of Radiology, University General Hospital “Attikon”, National and Kapodistrian University of Athens, 12462 Athens, Greece; alexop@med.uoa.gr (E.A.); argyromaz@med.uoa.gr (A.M.); 2 University Hospital of Heraklion, Medical School, University of Crete, 70013 Heraklion, Greece; raisakim@uoc.gr; 3 Pediatric Radiology Unit, Radiology Department, Hospital Universitario Virgen del Rocío, Avenida Manuel Siurot s/n, 41013 Seville, Spain; pablo.caro.sspa@juntadeandalucia.es; 4 Department of Pediatric Radiology, University Hospital, Liebigstraße 20a, 04107 Leipzig, Germany; franzwolfgang.hirsch@medizin.uni-leipzig.de; 5 Radiology Department, Faculty of Medicine, Institute for Children and Adolescents Health Care of Vojvodina, University of Novi Sad, 21000 Novi Sad, Serbia; jovan.lovrenski@mf.uns.ac.rs; 6 Department of Radiology and Nuclear Medicine, Erasmus MC—Sophia Children’s Hospital, 3015 CN Rotterdam, The Netherlands; p.ciet@erasmusmc.nl; 7 Department of Radiology, University of Cagliari, 09124 Cagliari, Italy

**Keywords:** community-acquired pneumonia, complicated pneumonia, pediatrics, ultrasound, computed tomography, magnetic resonance imaging

## Abstract

The most common acute infection and leading cause of death in children worldwide is pneumonia. Clinical and laboratory tests essentially diagnose community-acquired pneumonia (CAP). CAP can be caused by bacteria, viruses, or atypical microorganisms. Imaging is usually reserved for children who do not respond to treatment, need hospitalisation, or have hospital-acquired pneumonia. This review discusses the imaging findings for acute CAP complications and the diagnostic role of each imaging modality. Pleural effusion, empyema, necrotizing pneumonia, abscess, pneumatocele, pleural fistulas, and paediatric acute respiratory distress syndrome (PARDS) are acute CAP complications. When evaluating complicated CAP patients, chest radiography, lung ultrasonography, computed tomography (CT), and magnetic resonance imaging (MRI) can be used, with each having their own pros and cons. Imaging is usually not needed for CAP diagnosis, but it is essential for complicated cases and follow-ups. Lung ultrasound can supplement chest radiography (CR), which starts the diagnostic algorithm. Contrast-enhanced computed tomography (CECT) is used for complex cases. Advances in MRI protocols make it a viable alternative for diagnosing CAP and its complications.

## 1. Introduction

Pneumonia is one of the most common acute infections and the single most frequent cause of death in children worldwide, accounting for 14% of all deaths in children under 5 years of age [1,2]. Pneumonia killed 740,180 children under the age of 5 in 2019, accounting for 14% of all deaths of children under 5 years old but 22% of all deaths in children aged 1–5 years [2]. 

Community-acquired pneumonia (CAP) manifests as pneumonia symptoms, such as coughing, shortness of breath, and fever, in a previously healthy child due to an infection contracted outside of a hospital setting [3,4,5]. Laboratory findings useful for diagnosing CAP include the increase in erythrocyte sedimentation rate, white blood cell count, and C-reactive protein [6]. These markers are useful for monitoring treatment response [6].

The underlying causes can be bacterial, viral, or atypical (mycoplasma and chlamydia species). A significant proportion of severe pneumonia cases (up to 34%) result from mixed infections involving both viral and bacterial agents [7,8]. Although pneumonia’s causes include fungi and parasites, these are not common in the setting of CAP. Etiology can be presumed based on the patient’s age; children below 5 years have more frequent viral infections, while older children suffer from bacterial infections [3,4]. 

The predominant pathogens associated with CAP include the following: Streptococcus pneumoniae is the main etiological agent responsible for bacterial pneumonia in children. Haemophilus influenzae type b (Hib) is the second most prevalent etiological agent causing bacterial pneumonia. Respiratory syncytial virus is the predominant viral aetiology of pneumonia. Pneumocystis jiroveci is a prevalent cause of pneumonia among HIV-infected neonates, accounting for a minimum of 25% of pneumonia-related fatalities in this population [2]. Other bacterial pathogens appear to be less frequent causes of CAP. Group A streptococcal infection is important in terms of severity as, when present, it is more likely to progress to paediatric ICU admission or empyema [4]. Overall, viruses appear to account for 30–67% of CAP cases in childhood [9]. Mycoplasma is an etiological agent that is not unusual in ages 1–5 years old [4].

The diagnosis of CAP is based on clinical and laboratory findings, whereas imaging is not required in an immunocompetent child with uncomplicated CAP that does not need hospitalisation, since it will not change the management and outcome [3,4,10]. 

Imaging is mandatory when there is no response to outpatient treatment, in cases of hospital admission and in hospital-acquired pneumonia [10]. 

The potential acute complications of CAP can be pulmonary (pleural effusion, empyema, necrotizing pneumonia, lung abscess, bronchopleural fistula, pneumothorax, and acute respiratory failure), focal extrapulmonary (due to septicaemia and metastatic infection), and systemic (systemic inflammatory response syndrome or hemolytic uremic syndrome) [3]. 

When evaluating complicated CAP patients, chest radiography (CR), lung ultrasonography (LUS), computed tomography (CT), and magnetic resonance imaging (MRI) can be used, with each having their own pros and cons (Table 1). Imaging is usually not needed for CAP diagnosis, but it is essential for complicated cases and follow-ups.

The scope of this review is to present the imaging findings regarding the potential acute complications of CAP and to describe the role of each imaging modality. 

## 2. Imaging Modalities 

### 2.1. Chest Radiography 

Chest radiography (CR) is the most frequently performed imaging modality, which clinicians are more familiar with, for interpreting pathological CAP findings. For a proper assessment, several parameters should be considered, such as the technical parameters of the examination (kV and mAs), projection (AP versus PA), the rotation of the child, and the degree of inspiration. Although radiation exposure in CR is low, proper consideration should be given in order not to overexpose the child. Thus, the use of lateral CR views is usually avoided in the pediatric population for radiation safety, although the Paediatric Infectious Diseases Society and the Infectious Diseases Society of America recommend the use of both projections in their guidelines [3,11,12]. Special consideration should be given to hidden areas by other anatomical structures, such as the heart, the mediastinum, and the diaphragm [12]. Great variations in intra- and interobserver agreement between radiologists in CR interpretation are a limitation when using this modality [11]. Systematic reviews have calculated that the pooled sensitivity and specificity for pediatric pneumonia diagnosed by CXR can be up to 0.91 (95% CI: 0.90 to 0.93), 1.00 (95% CI: 0.99 to 1.00), and the pooled sensitivity and specificity for children with pneumonia diagnosed by LUS were 0.95 (95% CI: 0.94 to 0.96), 0.90 (95% CI: 0.87 to 0.92), respectively [13].

### 2.2. Lung Ultrasound 

Lung ultrasound (LUS) is a bedside modality that can be used when assessing a child with CAP. As a widely available, non-ionising, and low-cost technique, LUS is especially useful when assessing bed-ridden patients in the point-of-care ultrasound (POCUS) setting. This is a real-time examination where the examined child does not need to be sedated, allowing for a full examination of the chest. It is the best technique to evaluate the amount and characteristics of pleural effusion and the mobility of the diaphragm [14]. Rough estimates of the relative position of intrapulmonary lesions can also be obtained. LUS can detect small multifocal consolidations adherent to the subpleural surface [15,16]. Its main disadvantages include the reliance on operator skills for accurate results, the potential for time-intensive procedures (taking up to 20 min per examination), and the presence of hidden areas, like the subscapular or central lung regions, where a comprehensive assessment of parenchyma may not be achievable. Moreover, lesions that are not in touch with the pleura might be missed at the LUS examination [17,18,19,20]. If LUS is used to assess for CAP complications in the lung parenchyma, such as necrotizing areas or lung abscesses, Colour-Doppler (CD) and/or contrast-enhanced ultrasonography (CEUS) may be used [21,22]. Systematic reviews comparing CR and LUS suggest the excellent diagnostic accuracy of the latter, and that LUS is more suitable than CR for detecting pleural effusion, with LUS being able to detect effusions as small as 10 mL, whereas chest X-rays only identify larger effusions (200 mL) [23,24].

### 2.3. Computed Tomography 

Computed tomography (CT) is considered the gold-standard technique in terms of sensitivity in detecting CAP and its acute complications, but its use is limited due to the high radiation doses that are needed compared to CR. In the paediatric population, growth refers to an increase in cell proliferation, resulting in a greater number of cells undergoing mitosis and displaying heightened sensitivity to radiation. This is exacerbated by the fact that in children have a higher percentage of red bone marrow compared to adults, making them three times more radiation-sensitive compared to adults [25]. Advancements in technology, such as a new generation of scanners and the implementation of iterative reconstruction techniques, have made it possible to greatly decrease the radiation exposure during medical examinations. Achievable doses, measured in dose length products (DLPs), can now be as low as 7.66 mGy·cm per examination [26]. 

**Figure 1:**
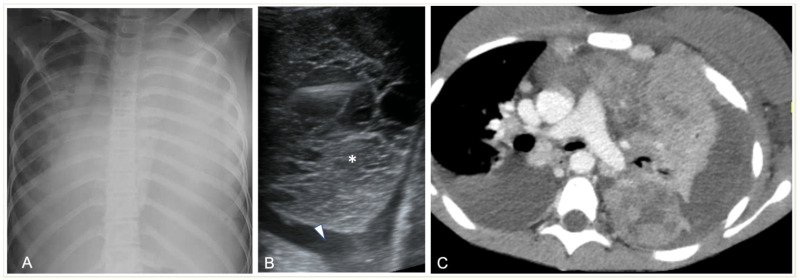
Imaging studies of a 14-year-old girl with respiratory distress, fever, and biopsy-proved non-Hodgkin Lymphoma. (**A**) Chest radiograph shows complete opacification of left hemithorax with mediastinal shift and some right lung opacities with pleural effusion. (**B**) LUS shows small left-sided pleural effusion (arrowhead) surrounding a lung mass (*) with multiple fluid-filled cavities. (**C**) Axial contrast-enhanced CT scan shows an inhomogeneously enhancing left lung mass with multiple necrotic areas, thoracic wall infiltration, bilateral pleural effusions, and enlarged mediastinal lymph nodes.

Previous generation scanners (the first generation and some of the second generation) required sedation in children younger than 4 years of age. However, third-generation scanners have overcome this problem, enabling high-speed scans with full acquisition of the thorax in less than a second without anaesthesia [27]. This technique is achieved using a dual-source scan with a maximum rotation time and a minimum pitch > 1.5. The use of an intravenous (IV) iodine contrast is mandatory when assessing for acute complications of CAP, such as pleural empyema and parenchymal necrosis. The examination protocol includes a single-phase scan directly after the IV contrast injection, without a non-contrast scan. In these complex clinical situations, contrast-enhanced CT enables image-guided intervention and serves as a pre-operative examination prior to video-assisted thoracoscopy (VATS) or in instances of diagnostic uncertainty (to rule out concurrent malignancy) (Figure 1). CT is suitable when the diagnosis of an abscess or necrotizing pneumonia cannot be confirmed with CR and LUS, in cases of extensive cavitation or loculated empyema, and to exclude the presence of a bronchopleural fistula. In cases where pleural involvement has to be assessed, the timing of the acquisition after intravenous contrast injection should be longer for the better enhancement of pleural folds. Lastly, CT is the technique of choice in children with recurrent pneumonia to determine the cause of recurrent infections, such as aspirated foreign bodies or possible congenital lung abnormalities [10,27,28,29]. 

### 2.4. Magnetic Resonance Imaging 

Magnetic Resonance Imaging (MRI) is a radiation-free technique that provides excellent soft-tissue contrast and anatomical detail. Still, it comes at a higher cost per examination and is less available in low-income countries. MRI is usually a more time-consuming examination than CT, with an increased need for sedation in children younger than 5 years. However, the recently reported use of real-time MRI for imaging pneumonia is just as fast as a CT scan, and it no longer requires sedation. Movement and breathing by the child do not cause artefacts either [30]. To date, this technique has only been available in research settings and is limited to a single MRI brand [31]. Spatial resolution of MRI is usually lower than CT, but new sequences, like ultra-short and zero echo time sequences, allow isotropic voxel sizes of around 1 mm [32]. In cooperative children, MRI has some advantages over CT. Firstly, MRI does not require the use of contrast to identify necrotic lung tissue from abscesses, which can be detected on T2-weighted images as hyperintense lung regions [33]. Secondly, MRI allows for the detection of septation on complex pleural effusions and empyema, which cannot be seen on CT. This allows for relevant information to be obtained in a single examination instead of using both LUS and CT. Finally, MRI can be repeated at the end of treatment as a follow-up technique to assess residual abnormalities and the effect of treatment. In the study of Konietzke et al., MRI showed similar results to CT in assessing necrotizing pneumonia and pulmonary abscess when both CR and LUS were inconclusive [34]. Contraindications to MRI may include unstable patients, claustrophobia, metallic implants, or a lack of cooperation [27,34,35,36,37]. The basic MRI protocol for lung examinations consists of T1- and T2-weighted sequences in at least two planes, diffusion-weighted (DWI) images, and post-contrast T1 weighted images with fat saturation.

#### 2.4.1. Pleural Effusion—Empyema 

A pleural effusion is formed when there is an imbalance between hydrostatic and oncotic pressure between the systemic and pulmonary circulations and the pleural space [38]. Additionally, the obstruction of lymphatic drainage by thick pleural fluid and debris further contributes to the accumulation of the effusion [6,39,40]. Parapneumonic effusion is a pleural effusion associated with an ipsilateral lung infection and can be simple or complicated (in the presence of infecting organisms). An empyema is a thick, purulent pleural effusion, which can be either free or loculated [41]. The progression of pleural effusion to empyema occurs in three stages: (a) the exudative stage, which is a simple parapneumonic effusion; (b) the fibrinopurulent stage, which is a complicated parapneumonic effusion; and (c) the organising phase, in which fibroblastic activity and peel formation occur. The distinction between transudate, exudate, and empyema is also based on chemical analysis. If a child is pyrexial or sick 48–72 h after beginning antibiotics, empyema may be present. Pneumonia and pleural effusion cause dullness to percussion and diminished breath sounds on physical examination. Consolidation increases fremitus. Detecting fremitus in children may be less helpful than detecting fremitus in adults due to age, size, and cooperation. Pleural effusion does not cause bronchial breathing [6].

**Figure 2:**
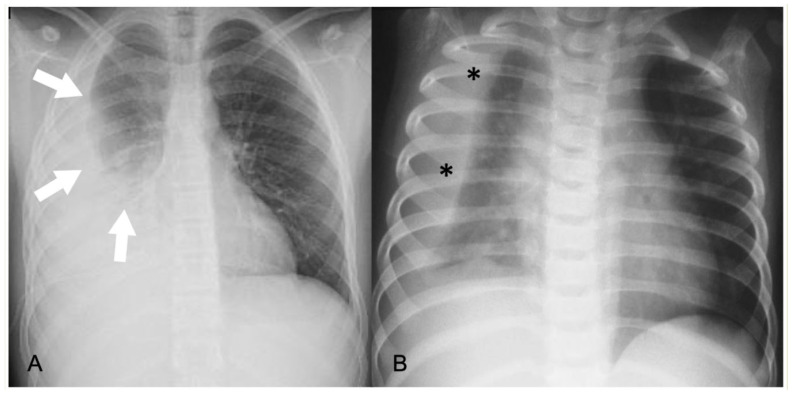
Pleural effusions on chest radiographs: (**A**) meniscus sign (arrows); (**B**) loculated effusion with convex borders (*) towards the spine.

In CR, pleural effusion may have different appearances, such as blunting of the costophrenic angle or the meniscus sign when free fluid is present (Figure 1). A medial convex border to the lung parenchyma indicates a loculated effusion. Complete opacification of the ipsilateral hemithorax is present in large effusions where the underlying parenchyma is obscured. An ipsilateral concave scoliosis and contralateral mediastinal shift may be common associated findings, and further imaging is required to investigate the condition of the underlying lung and pleura (Figure 2).

**Figure 3:**
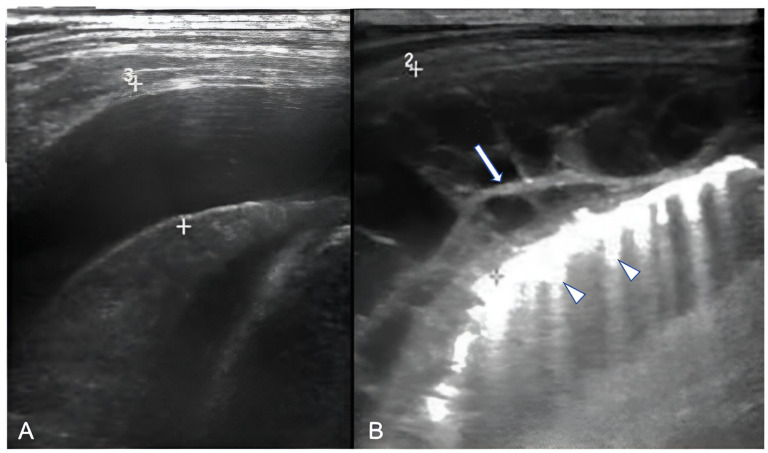
Ultrasonographic appearances of pleural effusions between cursors: (**A**) simple (anechoic), amenable for drainage under the appropriate clinical setting; (**B**) complicated, with internal septae (arrow). Also, note confluent B-lines (arrowheads).

LUS can provide information regarding the volume of the effusion (as small as 10 mL), the presence of fibrinous septations, and the echogenicity grade of the pleural fluid (Figure 3) [21]. LUS can be used to differentiate pleural effusions from consolidated lung and peripheral lung abscesses from empyema. In the appropriate clinical setting, the pulmonologist or thoracic surgeon may decide whether to perform thoracocentesis and put on a chest tube, fibrinolysis, or VATS based on the appearance and volume of the pleural effusion. The indication for performing thoracentesis arises when a pleural effusion, which may vary in volume, shows resistance to IV antibiotic treatment or when the patient continues to experience symptoms [42,43]. A few formulae can be used to calculate pleural effusion volume in adults; however, none of them are truly adapted to children. LUS also provides guidance for the insertion of draining catheters. Intravenous CEUS may be useful to better outline pleural effusions, whereas intracavitary contrast is helpful in determining catheter localization and patency, or the presence of loculation [20,44,45,46,47].

**Figure 4:**
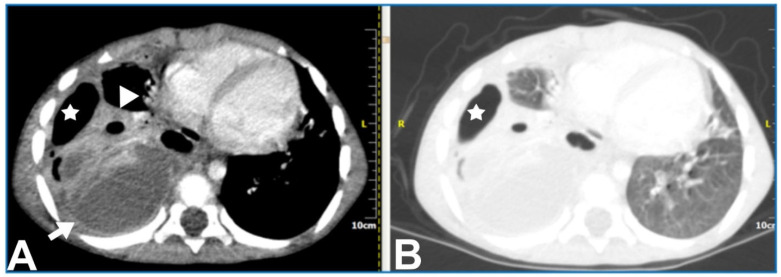
A 5.5-year-old boy with complicated pneumonia underwent a CT scan because of a differential diagnostic issue between empyema and lung abscesses on CR and LUS. Axial chest contrast-enhanced CT scan with soft-tissue window (**A**) and lung window (**B**) depicts a large multiloculated empyema (arrow), collapsed lung parenchyma with cavitary necrosis (arrowhead), and loculated pneumothorax (stars). Further, optimal treatment was drainage of the empyema based upon the chest CT findings.

Contrast-enhanced CT can demonstrate the location and volume of the effusion, although it might not show existing septations. The enhancement of thickened parietal pleura is usually depicted in this setting, although its absence does not exclude an infected effusion (Figure 4) [41].

**Figure 5:**
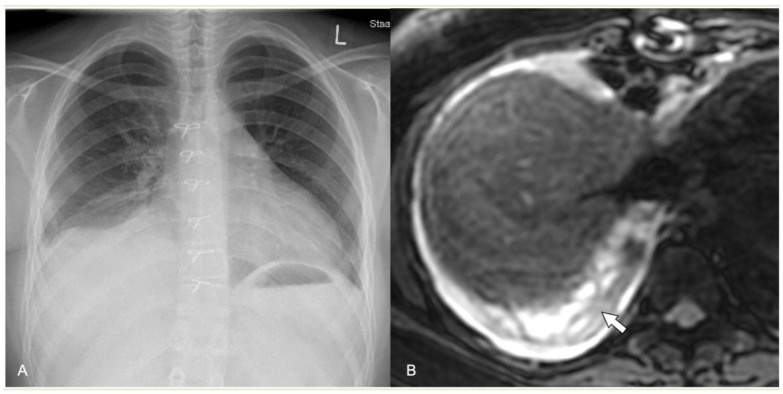
Complicated pneumonia following heart surgery. (**A**) Chest radiograph demonstrates the blunting of right pleurodiaphragmatic angle and a possible subpulmonary pleural effusion. (**B**) Axial MRI, T2-weighted sequence. Note the clear depiction of a pleural effusion with septations (arrow).

Regarding MRI, simple effusions show crescentic distribution and low signal on T1-weighted sequences, high signal on T2-weighted sequences, and no contrast enhancement. Empyema shows a loculated effusion with thickened, enhancing pleura, septations, and heterogeneous contents on T1-weighted and T2-weighted images due to debris (Figure 5). The use of DWI can help in differentiating between simple effusion and empyema, as the latter shows restricted diffusivity [27,48]. In a recent study, the use of intravenous contrast was equivocal as it provided no added value regarding the differentiation of pleural effusion and empyema, since all empyemas showed septations on T2-weighted sequences [34]. Differentiating fibrinous from non-fibrinous complex pleural effusions is one area where MR spectroscopy may be useful in everyday clinical practice, while its practicality is still debatable [49].

#### 2.4.2. Acute Lung Parenchyma Complications 

Three significant acute complications of severe pneumonia result from the extensive destruction and liquefaction of lung tissue caused by vascular thrombosis: necrotizing pneumonia, cavitary necrosis, and the formation of lung abscesses [6].

#### 2.4.3. Necrotizing Pneumonia

Children with necrotizing pneumonia exhibit symptoms such as fever, cough, and tachypnoea that last for many days. Hypoxia, moderate anaemia, and hypoalbuminaemia are frequent symptoms, with pleural effusion commonly visible upon physical examination [6].

**Figure 6:**
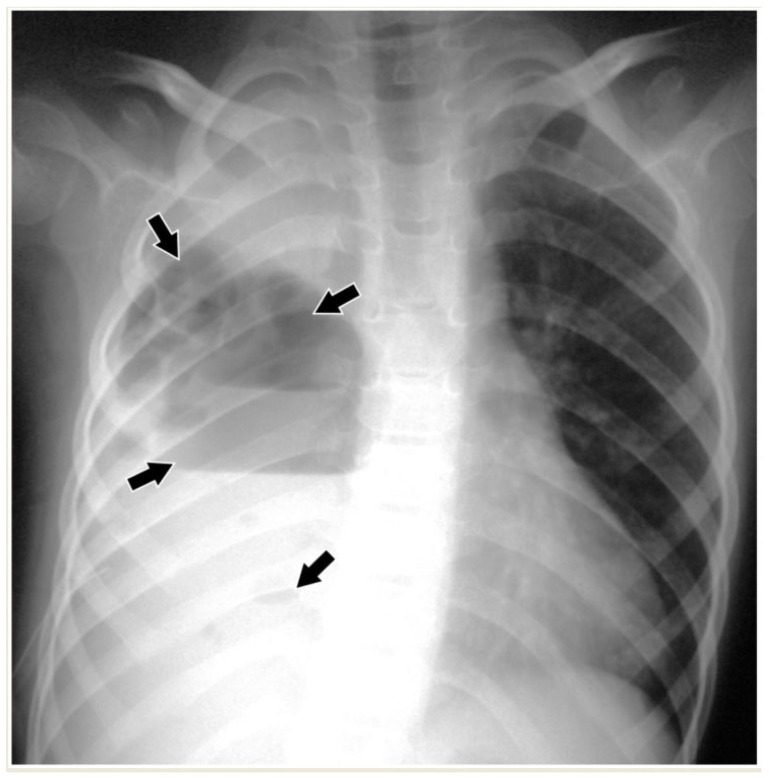
Necrotizing pneumonia in an 8-year-old boy. Chest radiograph shows opacification of the right hemithorax with ipsilateral concave scoliosis and multiple cavities (arrows) with air–fluid levels of variable size.

CR is not useful in the early stage of necrotizing pneumonia, as it is unable to depict liquefaction of the lung parenchyma. Cavitary necrosis typically exhibits the presence of air-filled cavities or air–fluid levels, which may require additional distinction from lung abscess or loculated empyema (Figure 6) [6,41].

**Figure 7:**
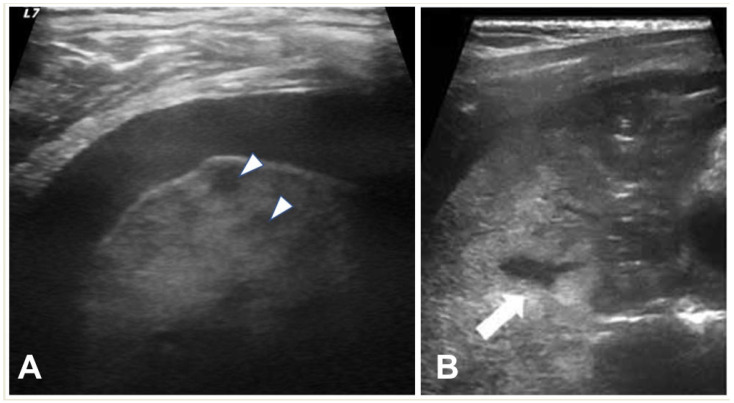
(**A**) LUS shows a consolidated lung with multiple hypoechoic areas (arrowheads) consistent with necrotising pneumonia in a 5.5-year-old boy. Pleural effusion is also present. (**B**) Necrotizing pneumonia in a 7-year-old girl; there is extensive consolidation containing hypoechoic areas with an ill-defined wall (arrow) consistent with necrosis.

**Figure 8:**
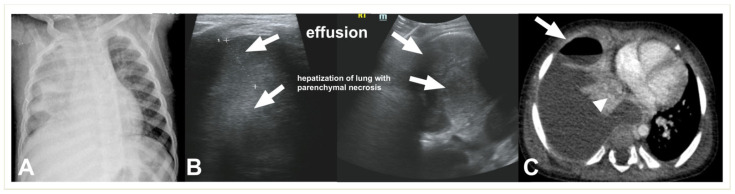
Complicated pneumonia in a 1-year-old boy. (**A**) Chest radiograph shows opacification of the right hemithorax with areas of consolidation and collapse. (**B**) Lung US shows an echogenic pleural effusion, making it difficult to distinguish from adjacent echogenic and necrotic lung parenchyma (**C**). Post-contrast CT scan images depict a large effusion, a loculated air–fluid level in the pleural space (thick arrow), and adjacent collapsed–consolidated lung parenchyma with necrotic areas (arrowhead).

Necrosis is seen on LUS as ill-defined areas of low echogenicity and diminished vascularity (Figure 7). Attention needs to be paid to the differentiation between echogenic effusions and echogenic lung parenchyma due to pneumonia (Figure 8). When positioned in the peripheral lung, LUS demonstrates comparable accuracy to CT in identifying pneumonia sequelae, such as pleural effusion, necrosis, and abscesses of lung parenchyma. However, the LUS sensitivity is lower than that of CT for centrally located abnormalities. The presence of fluid and static air bronchograms within a consolidated region may indicate the early onset of parenchymal complications [50]. The role of intravenous CEUS in complicated pneumonia remains a subject of controversy. While some researchers have reported on its accurate identification of necrotizing pneumonia and the precise delineation of its extent, contrasting findings have emerged from other authors, who found that no significant additional information was garnered in comparison to the utilisation of Colour-Doppler [20,22,45,46,51]. 

**Figure 9:**
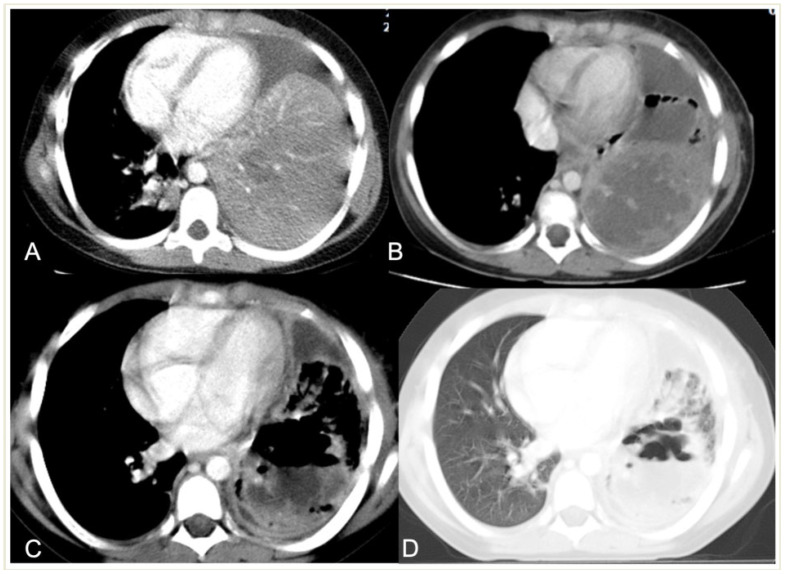
Development of necrotic and cavitary pneumonia in a 7-year-old girl. (**A**) There is a left basal consolidation with a central hypo-enhancing area, consistent with early necrosis. (**B**) Follow-up CT scan shows extensive areas of non-enhancement, consistent with extensive necrotic pneumonia. (**C**,**D**) Follow-up post-contrast scan, soft tissue, and lung window demonstrate cavitary necrosis containing multiple air–fluid levels.

Upon contrast-enhanced CT (CECT), necrotizing pneumonia is characterised by the presence of several patchy or widespread areas of diminished or absent enhancement, indicating liquefaction of the lung tissue (Figure 8). Cavitary necrosis is distinguished by the absence of a normal lung tissue structure, which is either diminished or lacking enhancement, and the appearance of many thin-walled cavities filled with either fluid or air. These cavities lack an enhancing border (Figure 9). 

**Figure 10:**
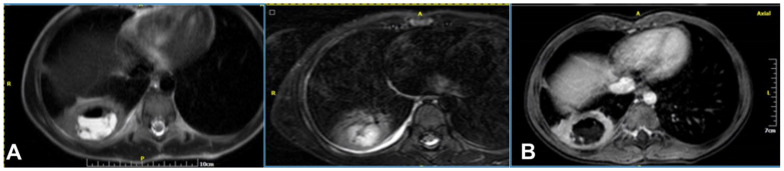
A 6-year-old boy, referred to MRI for exclusion of a lung abscess. (**A**) T2-weighted sequence shows a right lower lobe consolidation with a single air–fluid cavity (area with high and very low signal intensity), without a clearly depicted wall (area of isointensity surrounding the cavity) and an adjacent small pleural effusion (crescent-shaped line of high signal intensity). (**B**) Post-contrast T1-weighted sequence shows contrast enhancement by the adjacent consolidated lung parenchyma, consistent with the presence of cavitary necrosis.

Pulmonary necrosis on MRI is represented by areas of increased signal intensity on T2-weighed sequences, while cavitary necrosis is represented by fluid-filled and air-filled areas within a consolidation that do not enhance after the injection of contrast (Figure 10). Contrast-enhanced MRI shows a similar CECT pattern to identify necrotic lung parenchyma.

#### 2.4.4. Abscess

Children with lung abscesses often have an extended cough and mild fever, with less common symptoms including chest discomfort, dyspnoea, sputum production, and hemoptysis. A chest exam may show normal or symptoms of consolidation [6].

**Figure 11:**
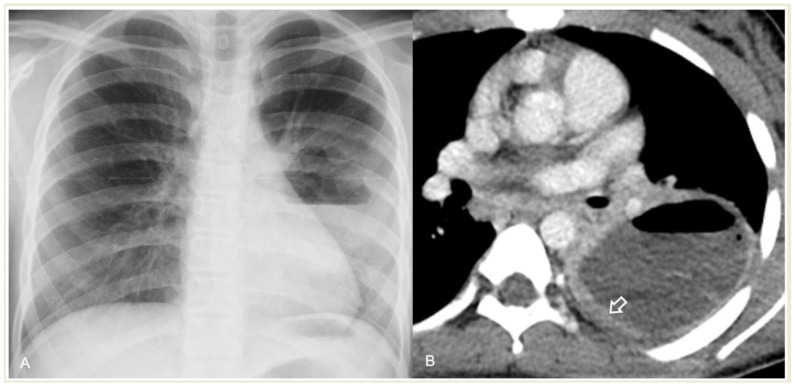
Lung abscess complicated CAP in a 12-year-old girl: (**A**) chest radiograph shows a cavity with an air–fluid level; (**B**) contrast-enhanced CT scan demonstrates a cavity with a thick enhancing wall (arrow) containing an air–fluid level, favoring the diagnosis of an abscess.

**Figure 12:**
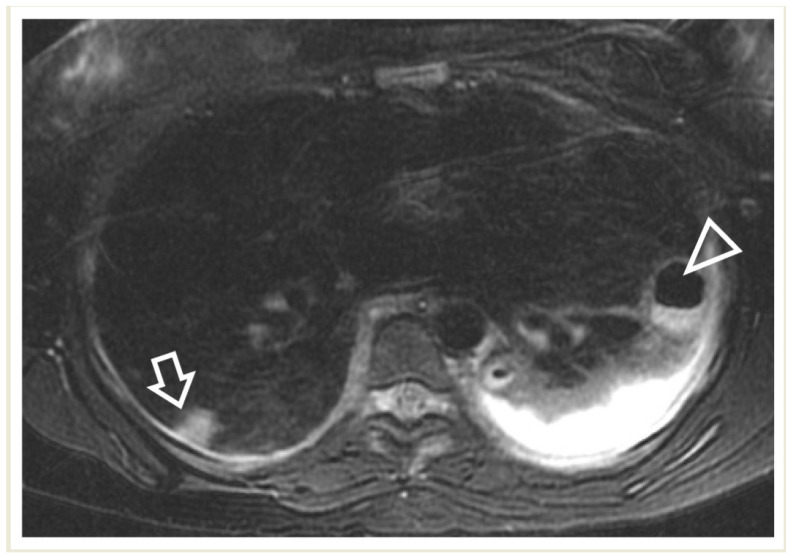
A 17-year-old girl with a pulmonary abscess following septic embolism. Lung MRI, in an axial T2-weighted image, shows areas of high signal intensity consistent with subpleural septic emboli (arrow) and pleural effusion (very high-signal-intensity area dorsally, mostly on the left side). Note the abscess at the left lung base, exhibiting a low signal and relatively thick wall, and containing an air–fluid level (arrowhead).

**Figure 13:**
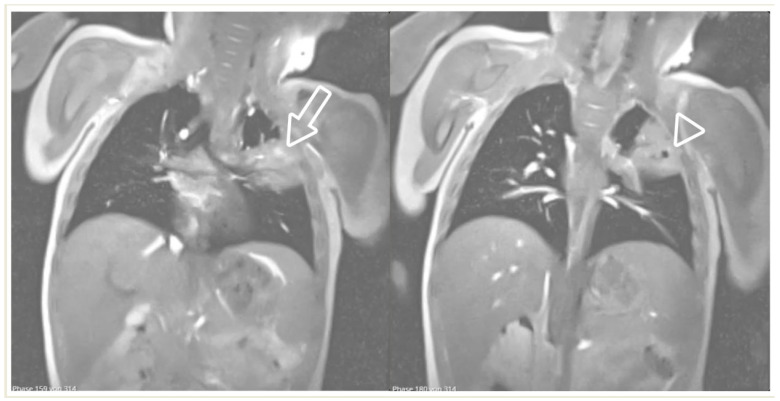
Real-time MRI sequence in the coronal plane in a 1-year-old girl with left upper-lobe pneumonia. Selected images from the free-breathing scan, which took 16 s to acquire and reconstruct (Supplementary Materials section). Within the isointense pulmonary infiltrate, there are areas of bright signal intensity (arrow) and a round area of very low (almost black) signal intensity, suggestive of air inclusions (arrowhead), with an appearance consistent with cavitary necrosis.

A lung abscess is a single cavity with a thick wall and purulent content resulting from suppuration and necrosis of the parenchyma. After the initial stage, the abscess can be partially or even completely air-filled. This is an uncommon complication in immunocompetent children with CAP but, when present, it may be associated with a pre-existing pulmonary cavity, such as cystic pulmonary adenomatoid malformations (CPAM). Severe forms of necrotizing pneumonia have also been associated with Staphylococcus aureus strains expressing the Panton–Valentine leukocidin cytotoxic agent [6,52]. A lung abscess on CR appears as a round or oval-shaped area that is denser than the surrounding lung parenchyma. The edges of the abscess might be irregular or ill-defined, and there may be air–fluid levels inside, indicating the presence of gas and fluid. On LUS, a lung abscess typically appears as a hypoechoic area within the lung tissue with irregular borders. Surrounding the hypoechoic area, there might be a hyperechoic rim, which corresponds to the inflamed tissue. On CECT, this appears as a hypodense cavity surrounded by a well-defined enhancing wall without central enhancement and a variable proportion of air and/or fluid content (Figure 11). On MRI, a rounded, T2-hyperintense area surrounded by a hypointense wall can be seen, which is usually thick and irregular and may contain an air–fluid level or be completely air-filled (Figure 12 and Figure 13 and Supplementary Material). A characteristic, restricted diffusion can be seen in DWI sequences, with peripheral wall enhancement after the injection of intravenous contrast [27,34,48,53,54,55].

#### 2.4.5. Pneumatocele

A pneumatocele is a thin-walled intraparenchymal cystic structure that is filled with air (or little-to-no fluid content) and is formed by a valve-type mechanism that allows for the one-way passage of air into the interstitial space. Pneumatocele are often the results of severe infections, trauma, and mechanical ventilation. Pneumatocele appear as bubble-like structures on imaging studies like X-rays or CT scans [56]. Pneumatocele are difficult to identify on LUS because they are usually filled with air and therefore have an anechoic appearance.

#### 2.4.6. Pleural Fistulas 

**Figure 14:**
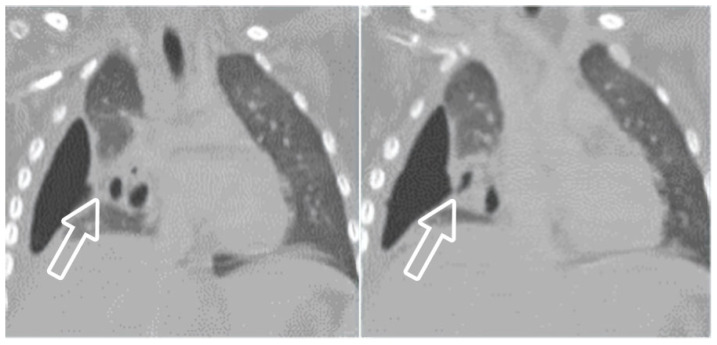
A 4-year-old girl with complicated pneumonia and a surgically confirmed broncho-pleural fistula. CT scan and coronal reconstruction show right loculated pneumothorax and collapsed lung parenchyma, and suggest the fistula to be an indentation (arrow) at the visceral pleura adjacent to a pneumatocele.

A pleural fistula can form during severe pneumonia as a result of the erosion or perforation of lung tissue that connects the bronchi to the pleural space. Pleural fistula is a serious and potentially life-threatening complication in severe pneumonia, leading to respiratory distress, sepsis, and pneumothorax. The diagnosis of bronchopleural fistula is usually definite on CT when a direct communication between the lung parenchyma and the pleural space is identified. It is also suspected when there is a persistent air leak in the pleural space for more than 24 h (Figure 14) or when pneumothorax recurs following the removal of the drains [57]. CECT is the optimal technique for diagnosing a pleural fistula due to its high resolution, which enables the identification of direct connections between small peripheral bronchi and the pleural space [58].

#### 2.4.7. Pediatric Acute Respiratory Distress Syndrome (PARDS)

**Figure 15:**
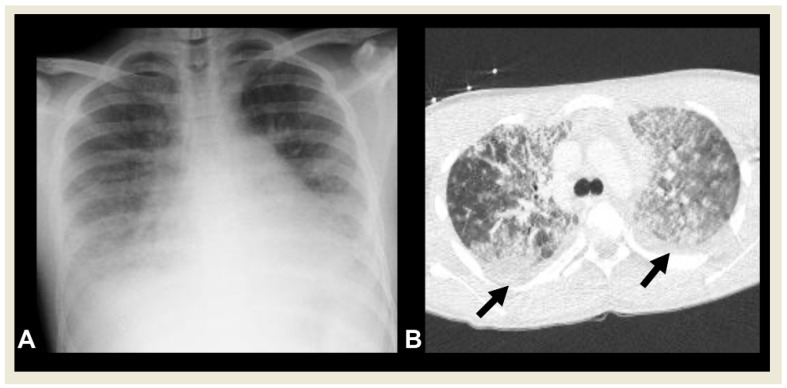
A 15-year-old boy with PARDS. (**A**) Chest radiography shows bilateral, diffuse lower lung opacities, and pleural effusions. (**B**) CECT shows bilateral ground-glass and alveolar opacities, and interlobular septal thickening with concomitant pleural effusions (arrows).

PARDS is defined as the occurrence of signs and symptoms of pulmonary oedema that are not fully explained by cardiac failure or fluid overload within 7 days of a clinical insult [59]. Various epidemiological factors and etologies play a significant role in the development of PARDS. The most common PARDS risk factors include pneumonia or lower respiratory tract infections [60]. One of the clinical and laboratory criteria used to diagnose PARDS is the detection of bilateral lung infiltrates on chest radiography (Figure 15) [59].

## 3. Conclusions

Imaging is usually not needed for the initial diagnosis of CAP; however, it becomes crucial for identifying acute complications and for follow-up assessments. The diagnostic algorithm always starts with CR which can be supplemented with LUS, particularly to confirm the presence of pleural effusion and empyema. In cases without severe complications, the combined findings from these two techniques are sufficient for patient management.

CECT scanning is reserved for more complicated cases, enabling the assessment of severe parenchymal abnormalities, such as necrotizing pneumonia, cavitary lesions, abscesses, and pleural fistulas, or when surgical intervention is being considered. In skilled hands, LUS can offer valuable insights into complicated pneumonias, potentially obviating the need for more advanced diagnostic modalities like CT. Finally, emerging advancements in MRI protocols, such as real-time MRI and ultra-short and zero echo time sequences, as presented above, have positioned MRI as a viable alternative for the diagnosis of CAP and its complications, particularly in centres equipped with specialized expertise and resources. A suggested imaging algorithm, according to clinical indications, can be found in Table 2.

## Figures and Tables

**Table 1:** Imaging modalities’ pros and cons.

Modality	Pros	Cons
CR	Widely available, very quick examination	Radiation exposure, prone to misinterpretation
LUS	Widely available, no ionizing radiation, point-of-care examination	User-dependent and time-consuming examination, findings are dependent on the location
CT	Widely available, quick examination; excellent spatial resolution for chest examinations, with high sensitivity	Radiation exposure, patient cooperation needed
MRI	Excellent soft tissue contrast, no ionizing radiation	Not widely available, patient cooperation needed, time-consuming examination, newer techniques and software needed for lung examinations to improve spatial and temporal resolution

**Table 2:** Suggested imaging algorithm according to clinical indication.

Complicated CAP							Uncomplicated CAP
Pleural Effusion	Empyema	Necrotising Pneumonia	Abscess	Pneumatocele	Pleural Fistula	PARDS	
US: usually appropriate	US/CT: usually appropriate	US/CT: usually appropriate	US/CT: usually appropriate	CT: usually appropriate	CT: usually appropriate	CR: usually appropriate	No imaging is necessary
CR/CT: may be appropriate	MRI: may be appropriate	MRI: may be appropriate	MRI: may be appropriate	CR: may be appropriate		US/CT: may be appropriate	
MRI: usually not appropriate	CR: usually not appropriate	CR: usually not appropriate	CR: usually not appropriate	US/MRI: usually not appropriate	CR/US/MRI: usually not appropriate	MRI: usually not appropriate	

## Data Availability

No new data were created or analyzed in this study. Data sharing is not applicable to this article.

## Supplementary Materials

The following supporting information can be downloaded at: https://www.mdpi.com/article/10.3390/children11010122/s1, Video S1: Real-time MRI sequence in the coronal plane in a 1-year-old girl with left upper lobe pneumonia with an acquisition time of 16 s.

## References

[1] Wardlaw T., Salama P., Johansson E.W., Mason E. (2006). Pneumonia: The leading killer of children. Lancet.

[2] Pneumonia in Children. https://www.who.int/news-room/fact-sheets/detail/pneumonia.

[3] Bradley J.S., Byington C.L., Shah S.S., Alverson B., Carter E.R., Harrison C., Kaplan S.L., Mace S.E., McCracken G.H., Moore M.R. (2011). The Management of Community-Acquired Pneumonia in Infants and Children Older than 3 Months of Age: Clinical Practice Guidelines by the Pediatric Infectious Diseases Society and the Infectious Diseases Society of America. Clin. Infect. Dis..

[4] Harris M., Clark J., Coote N., Fletcher P., Harnden A., Mckean M., Thomson A. (2011). British Thoracic Society guidelines for the management of community acquired pneumonia in children: Update 2011. Thorax.

[5] Edis E.C., Hatipoglu O.N., Yilmam I., Eker A., Tansel O., Sut N. (2009). Hospital-Acquired Pneumonia Developed in Non-Intensive Care Units. Respiration.

[6] De Benedictis F.M., Kerem E., Chang A.B., Colin A., Zar H.J., Bush A. (2020). Complicated pneumonia in children. Lancet.

[7] Roh E.J., Shim J.Y., Chung E.H. (2022). Epidemiology and surveillance implications of community-acquired pneumonia in children. Clin. Exp. Pediatr..

[8] Von Mollendorf C., Berger D., Gwee A., Duke T., Graham S.M., Russell F.M., Mulholland E.K. (2022). ARI review group Aetiology of childhood pneumonia in low- and middle-income countries in the era of vaccination: A systematic review. J. Glob. Health.

[9] Cilla G., Oñate E., Perez-Yarza E.G., Montes M., Vicente D., Perez-Trallero E. (2008). Viruses in community-acquired pneumonia in children aged less than 3 years old: High rate of viral coinfection. J. Med. Virol..

[10] Chan S.S., Kotecha M.K., Rigsby C.K., Iyer R.S., Alazraki A.L., Anupindi S.A., Bardo D.M., Brown B.P., Chandra T., Dorfman S.R. (2020). ACR Appropriateness Criteria^®^ Pneumonia in the Immunocompetent Child. J. Am. Coll. Radiol..

[11] Cherian T., Mulholland E.K., Carlin J.B., Ostensen H., Amin R., De Campo M., Greenberg D., Lagos R., Lucero M., Madhi S.A. (2005). Standardized interpretation of paediatric chest radiographs for the diagnosis of pneumonia in epidemiological studies. Bull. World Health Organ..

[12] Andronikou S., Lambert E., Halton J., Hilder L., Crumley I., Lyttle M.D., Kosack C. (2017). Guidelines for the use of chest radiographs in community-acquired pneumonia in children and adolescents. Pediatr. Radiol..

[13] Yan J.-H., Yu N., Wang Y.-H., Gao Y.-B., Pan L. (2020). Lung ultrasound vs chest radiography in the diagnosis of children pneumonia. Medicine.

[14] Ibitoye B.O., Idowu B.M., Ogunrombi A.B., Afolabi B.I. (2018). Ultrasonographic quantification of pleural effusion: Comparison of four formulae. Ultrasonography.

[15] Brogna B., Bignardi E., Brogna C., Volpe M., Lombardi G., Rosa A., Gagliardi G., Capasso P.F.M., Gravino E., Maio F. (2021). A Pictorial Review of the Role of Imaging in the Detection, Management, Histopathological Correlations, and Complications of COVID-19 Pneumonia. Diagnostics.

[16] Kogias C., Prountzos S., Alexopoulou E., Douros K. (2023). Lung ultrasound systematic review shows its prognostic and diagnostic role in acute viral bronchiolitis. Acta Paediatr..

[17] Liu J., Copetti R., Sorantin E., Lovrenski J., Rodriguez-Fanjul J., Kurepa D., Feng X., Cattaross L., Zhang H., Hwang M. (2019). Protocol and Guidelines for Point-of-Care Lung Ultrasound in Diagnosing Neonatal Pulmonary Diseases Based on International Expert Consensus. J. Vis. Exp..

[18] Bhalla D., Naranje P., Jana M., Bhalla A.S. (2022). Pediatric lung ultrasonography: Current perspectives. Pediatr. Radiol..

[19] Lovrenski J. (2020). Pediatric lung ultrasound cons—Are they really strong enough?. Pediatr. Radiol..

[20] Lovrenski J. (2020). Pediatric lung ultrasound—Pros and potentials. Pediatr. Radiol..

[21] Laya B.F., Concepcion N.D.P., Garcia-Peña P., Naidoo J., Kritsaneepaiboon S., Lee E.Y. (2022). Pediatric Lower Respiratory Tract Infections: Imaging Guidelines and Recommendations. Radiol. Clin. N. Am..

[22] Dietrich C.F., Buda N., Ciuca I.M., Dong Y., Fang C., Feldkamp A., Jüngert J., Kosiak W., Mentzel H.J., Pienar C. (2021). Lung ultrasound in children, WFUMB review paper (part 2). Med. Ultrason..

[23] Chidini G., Raimondi F. (2023). Lung ultrasound for the sick child: Less harm and more information than a radiograph. Eur. J. Pediatr..

[24] Hansell L., Milross M., Delaney A., Tian D.H., Ntoumenopoulos G. (2021). Lung ultrasound has greater accuracy than conventional respiratory assessment tools for the diagnosis of pleural effusion, lung consolidation and collapse: A systematic review. J. Physiother..

[25] Nagy E., Tschauner S., Schramek C., Sorantin E. (2023). Paediatric CT made easy. Pediatr. Radiol..

[26] Worrall M., Holubinka M., Havariyoun G., Hodgson K., Edyvean S., Holroyd J., Davis A., Dunn M., Gardiner A. (2022). Analysis and results from a UK national dose audit of paediatric CT examinations. Br. J. Radiol..

[27] Liszewski M.C., Görkem S., Sodhi K.S., Lee E.Y. (2017). Lung magnetic resonance imaging for pneumonia in children. Pediatr. Radiol..

[28] Jaffe A., Calder A.D., Owens C.M., Stanojevic S., Sonnappa S. (2008). Role of routine computed tomography in paediatric pleural empyema. Thorax.

[29] Odev K., Guler I., Altinok T., Pekcan S., Batur A., Ozbiner H. (2013). Cystic and Cavitary Lung Lesions in Children: Radiologic Findings with Pathologic Correlation. J. Clin. Imaging Sci..

[30] Hirsch F.W., Sorge I., Vogel-Claussen J., Roth C., Gräfe D., Päts A., Voskrebenzev A., Anders R.M. (2020). The current status and further prospects for lung magnetic resonance imaging in pediatric radiology. Pediatr. Radiol..

[31] Hirsch F.W., Sorge I., Voit D., Frahm J., Prenzel F., Wachowiak R., Anders R., Roth C., Gräfe D. (2023). Chest examinations in children with real-time magnetic resonance imaging: First clinical experience. Pediatr. Radiol..

[32] Wielpütz M.O., Triphan S.M.F., Ohno Y., Jobst B.J., Kauczor H.-U. (2019). Outracing Lung Signal Decay—Potential of Ultrashort Echo Time MRI. RoFo Fortschritte Auf Dem Geb. Der Rontgenstrahlen Und Der Bildgeb. Verfahr..

[33] Liszewski M.C., Ciet P., Winant A.J., Lee E.Y. (2023). Magnetic Resonance Imaging of Pediatric Lungs and Airways. J. Thorac. Imaging.

[34] Konietzke P., Mueller J., Wuennemann F., Wagner W.L., Schenk J.-P., Alrajab A., Kauczor H.-U., Stahl M., Mall M.A., Wielpütz M.O. (2020). The value of chest magnetic resonance imaging compared to chest radiographs with and without additional lung ultrasound in children with complicated pneumonia. PLoS ONE.

[35] Peltola V., Ruuskanen O., Svedström E. (2008). Magnetic resonance imaging of lung infections in children. Pediatr. Radiol..

[36] Attenberger U., Morelli J., Henzler T., Buchheidt D., Fink C., Schoenberg S., Reichert M. (2014). 3Tesla proton MRI for the diagnosis of pneumonia/lung infiltrates in neutropenic patients with acute myeloid leukemia: Initial results in comparison to HRCT. Eur. J. Radiol..

[37] Sodhi K.S., Khandelwal N., Saxena A.K., Singh M., Agarwal R., Bhatia A., Lee E.Y. (2016). Rapid lung MRI in children with pulmonary infections: Time to change our diagnostic algorithms. J. Magn. Reson. Imaging.

[38] Calder A., Owens C.M. (2009). Imaging of parapneumonic pleural effusions and empyema in children. Pediatr. Radiol..

[39] Feller-Kopman D., Light R. (2018). Pleural Disease. N. Engl. J. Med..

[40] Lai-Fook S.J. (2004). Pleural Mechanics and Fluid Exchange. Physiol. Rev..

[41] Eslamy H.K., Newman B. (2011). Pneumonia in Normal and Immunocompromised Children: An Overview and Update. Radiol. Clin. N. Am..

[42] Tracy M.C., Mathew R. (2018). Complicated pneumonia: Current concepts and state of the art. Curr. Opin. Pediatr..

[43] Fischer G.B., Mocelin H.T., Andrade C.F., Sarria E.E. (2018). When should parapneumonic pleural effusions be drained in children?. Paediatr. Respir. Rev..

[44] James C.A., Lewis P.S., Moore M.B., Wong K., Rader E.K., Roberson P.K., Ghaleb N.A., Jensen H.K., Pezeshkmehr A.H., Stroud M.H. (2022). Efficacy of standardizing fibrinolytic therapy for parapneumonic effusion. Pediatr. Radiol..

[45] Deganello A., Rafailidis V., Sellars M.E., Ntoulia A., Kalogerakou K., Ruiz G., Cosgrove D.O., Sidhu P.S. (2017). Intravenous and Intracavitary Use of Contrast-Enhanced Ultrasound in the Evaluation and Management of Complicated Pediatric Pneumonia. J. Ultrasound Med..

[46] Tomà P. (2020). Lung ultrasound in pediatric radiology—Cons. Pediatr. Radiol..

[47] Chen H.-J., Yu Y.-H., Tu C.-Y., Chen C.-H., Hsia T.-C., Tsai K.-D., Shih C.-M., Hsu W.-H. (2009). Ultrasound in Peripheral Pulmonary Air-Fluid Lesions. Chest.

[48] Kapur S., Bhalla A.S., Jana M. (2019). Pediatric Chest MRI: A Review. Indian J. Pediatr..

[49] Chiu C.-Y., Cheng M.-L., Wong K.-S., Lai S.-H., Chiang M.-H., Tsai M.-H., Lin G. (2019). Metabolomics Reveals Anaerobic Bacterial Fermentation and Hypoxanthine Accumulation for Fibrinous Pleural Effusions in Children with Pneumonia. J. Proteome Res..

[50] Musolino A.M., Tomà P., Supino M.C., Scialanga B., Mesturino A., Scateni S., Battaglia M., Pirozzi N., Bock C., Buonsenso D. (2019). Lung ultrasound features of children with complicated and noncomplicated community acquired pneumonia: A prospective study. Pediatr. Pulmonol..

[51] Rafailidis V., Andronikou S., Mentzel H.-J., Piskunowicz M., Squires J.H., Barnewolt C.E. (2021). Contrast-enhanced ultrasound of pediatric lungs. Pediatr. Radiol..

[52] Eber Fabio E.M. (2021). ERS Handbook of Paediatric Respiratory Medicine.

[53] Gorkem S.B., Coskun A., Yikilmaz A., Zurakowski D., Mulkern R.V., Lee E.Y. (2013). Evaluation of Pediatric Thoracic Disorders: Comparison of Unenhanced Fast-Imaging-Sequence 1.5-T MRI and Contrast-Enhanced MDCT. Am. J. Roentgenol..

[54] Baez J.C., Ciet P., Mulkern R., Seethamraju R.T., Lee E.Y. (2015). Pediatric Chest MR Imaging. Magn. Reson. Imaging Clin. N. Am..

[55] Ciet P., Tiddens H.A.W.M., Wielopolski P.A., Wild J.M., Lee E.Y., Morana G., Lequin M.H. (2015). Magnetic resonance imaging in children: Common problems and possible solutions for lung and airways imaging. Pediatr. Radiol..

[56] Al-Saleh S., Grasemann H., Cox P. (2008). Necrotizing Pneumonia Complicated by Early and Late Pneumatoceles. Can. Respir. J..

[57] Andronikou S., Goussard P., Sorantin E. (2017). Computed tomography in children with community-acquired pneumonia. Pediatr. Radiol..

[58] Kapoor H., Gulati V., Gulati A., Donuru A., Parekh M. (2022). Comprehensive Imaging Review of Pleural Fistulas from Diagnosis to Management. RadioGraphics.

[59] Shein S.L., Maddux A.B., Klein M.J., Bhalla A., Briassoulis G., Dahmer M.K., Emeriaud G., Flori H.R., Gedeit R., Ilia S. (2022). Epidemiology and Outcomes of Critically Ill Children at Risk for Pediatric Acute Respiratory Distress Syndrome: A Pediatric Acute Respiratory Distress Syndrome Incidence and Epidemiology Study. Crit. Care Med..

[60] Kohne J.G., Flori H.R. (2019). Risk Factors and Etiologies of Pediatric Acute Respiratory Distress Syndrome. Pediatric Acute Respiratory Distress Syndrome.

